# TRAM: The Telecommunications-Related AcciMap Method

**DOI:** 10.1007/s42979-025-04502-3

**Published:** 2026-01-13

**Authors:** Hans C. A. Wienen, Faiza A. Bukhsh, Eelco Vriezekolk, Luís Ferreira Pires

**Affiliations:** 1 Faculty of Electrical Engineering, Mathematics and Computer Science, University of Twente, Drienerlolaan 5, 7522 NB Enschede, Overijssel The Netherlands; 2 Dutch Authority for Digital Infrastructure, Postbus 450, 9700 AL Groningen, The Netherlands

**Keywords:** Telecommunications, Accident analysis, Incident analysis, Cyber incidents, AcciMap method

## Abstract

Telecommunications networks are essential to the functioning of modern society, and large-scale disruptions in these networks can significantly impact societal operations. This paper presents Tram (Telecommunications Related AcciMap), an accident analysis method developed to support the analysis of accidents in telecommunication networks. Tram aimed to allow lessons learned from an accident to be used to mitigate and/or prevent new accidents, ultimately enhancing societal resilience. Initially, we studied the state-of-the-art in accident analysis methods, concluding that we should start from the AcciMap method since this is the most popular and appropriate method for our purposes. Tram has been developed through iterative cycles in which improvements have been proposed and validated; this paper focuses on the last iteration. Tram is a validated method that supports comprehensive analyses of telecom accidents. The latest version added support to the representation and mitigation of positive feedback loops during telecom and information technology systems breakdowns, and can help prioritise the recommendations derived from an analysis. This paper demonstrates that Tram is a suitable method to analyse and learn lessons from accidents in the telecom domain. Particularly, our findings with the application of Tram to real-life accidents indicate that dividing the analysis process by participant expertise can negatively impact the efficiency of the overall process, so the partitioning of the analysis process should be carefully considered.

## Introduction

Modern society is heavily dependent on telecommunications networks. These networks form the foundation of critical infrastructure that requires stability and resilience to ensure the normal functioning of societal processes. Incidents should be avoided, and when they happen, their consequences should be remedied as quickly as possible. Therefore, we learn from past incidents to gain insights that can prevent future incidents and learn how to remediate them, thereby improving the stability and resilience of telecommunications networks.

In literature on incident and accident analysis, there is a general consensus that the difference between an incident and an accident is whether or not the event caused damage [1], where an incident causes no damage (also called a *near miss*) and an accident causes damage. However, in the telecommunications domain, the term ‘incident’ is also used to indicate events that cause damage. Therefore, to align with broader accident analysis terminology and to avoid confusion, in this paper, we consistently use the term ‘accident’ to denote these hazardous events.

Our research focuses on developing an accident analysis method that meets the stringent requirements of the telecommunications sector. For this purpose, we have developed Tram (Telecom Related AcciMap), which enables the modelling and analysis of the distinctive features associated with accidents in telecommunications, possibly as a result of cyber attacks [2–4]. Tram has been developed in real-world corporate settings, characterised by time constraints and limited collaboration opportunities in which these companies operate, so controlled experiments were ruled out. To address this limitation, we applied TAR (Technical Action Research) [5], which enabled us to identify new issues during testing and validation of our accident analysis method. In each iteration, our method was applied in an uncontrolled environment, and as validation progressed, it was adapted to handle unexpected issues encountered during those validations, yielding an improved version of the method.

This paper extends our former publication [4] by updating the literature review reported in [1], and discussing in much more detail how Tram has evolved to its current version. Moreover, we discuss how Tram has been developed as an extension of the AcciMap method, by discussing Tram’s iterative development process. In [3], we identified that positive feedback loops can play a crucial role in telecommunications accidents. This paper then concentrates on the last iteration of the Tram’s development process, in which the modelling of positive feedback loops is validated, and the prioritisation of recommendations has been added to the method. The latter is particularly relevant for applying Tram in realistic scenarios since the method can yield a large number of recommendations (generally hundreds) and the former version of the method did not give guidelines on how to choose amongst them. Moreover, we emphasise the practical usability of the method by considering stakeholder needs, prioritising recommendations, and addressing diverse validation perspectives.

This paper is further structured as follows: Section Accident Analysis Methods discusses accident analysis methods, Section Tram describes the Tram method, Section Validation of Tram 2 describes the accident we used to improve and validate Tram in the last iteration, and presents the results of Tram’s application to this accident, Section Discussion discusses these results, and Section Final Remarks concludes this paper.

## Accident Analysis Methods

In the literature we could find three different families of accident analysis methods [6–8]:

*Sequential methods*: in which accidents are represented as the outcome of a sequence of events.
*Epidemiological methods*: in which accidents are represented as the outcome of a sequence of events that could take place in an environment, while the measures that should have inhibited this sequence of events were malfunctioning or missing. These methods take the socio-technical context into consideration, also considering aspects such as company culture, risk management, budgeting choices and safety regulations.
*Systemic methods*: in which not only the socio-technical context is taken into account, but also an attempt is made to model the system where the accident has taken place. These methods consider the tight links between parts of the system, positive and negative feedback loops, and discrepancies between the mental model based on which operators make decisions and the physical reality that they are influencing.

We have also identified some methods that do not fall into the families mentioned above, but these methods get limited attention in the literature and are thus less relevant [8].

The three families of methods mentioned above have been developed in chronological order. Sequential methods were developed first, with the Fault Tree Analysis method [9] as an example. The relevance of the socio-technical context became apparent in the 1990s, after, amongst others, the Bhopal disaster, the Challenger explosion and the Chernobyl nuclear disaster [10]. Consequently, around this time the first epidemiological methods were developed [11]. System-theoretical aspects were introduced in the 2000s, resulting in the development of systemic methods, such as AcciMap [11], Stamp [12] and Fram [7]. These methods evolved as a consequence of the occurrence of new accidents that could not satisfactorily be analysed with the methods available at that time. Systemic methods started to emerge after software got a more prominent role in both the design and the running of systems, thereby creating new vulnerabilities and sources for disasters [12].

**Fig. 1:**
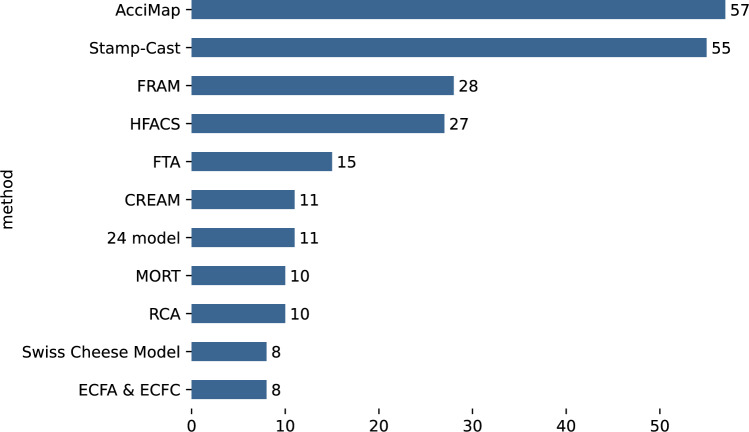
Top 11 of accident analysis methods ranked by the number of articles defining, applying and comparing them

In this work, we updated our literature review from 2015 [1], confirming the trends already apparent in 2015. From this update we conclude that accident analysis methods in academic literature are now dominated by systemic methods. Figure 1 shows that the three main methods of the systemic family of accident analysis methods (which are Stamp-Cast, Fram and AcciMap), AcciMap is the method on which most papers have been published in the literature. From our 2015 literature review [1] we concluded that AcciMap was the most appropriate method to base the development of our own method (Tram) upon, and this choice has been confirmed by the current state-of-the-art. The work of [13] also supported our findings that Accimap is one of the most used methods for accident analysis. Similar work by [14] indicated that among many, Accimap is one of the methods to analyse incidents, especially the construction incidents. Here, our work is different because we focused on IT-focused incidents in the telecom domain.

## Tram

This section discusses how Tram has evolved from AcciMap in iterative development steps, and presents some critical capabilities currently incorporated in the method.

### Evolution from AcciMap

We initiated our Technical Action Research [5] by seeking an accident analysis method that could facilitate the analysis of specific issues in telecommunications. We could not find any suitable method [1], so we selected an accident analysis method (AcciMap) that had been thoroughly applied in practice and could be extended to suit our purposes. We set out to test AcciMap in the telecommunications domain, and we selected as a starting point the *Generic AcciMap Method*  which was proposed by Branford [15] as a way to apply AcciMap and to make it practically useful. In essence, when applying the *Generic AcciMap Method* experts produce a graph that maps causes and consequences to a set of layers representing (*i*) the organisation in which the accident to be analysed occurred, (*ii*) the external world, (*iii*) the physical world in which actors perform actions, and (*iv*) the outcomes, respectively. This graph is represented in a so-called *AcciMap diagram*, which acts as an instrument for analysing the accident during planned workshops with the participation of company experts.

#### First Iteration

We applied the *Generic AcciMap Method* to a case study with a Western European telecommunications company, to analyse an accident consisting of a complete service outage caused by a ddos-attack [2]. When applying the *Generic AcciMap Method* to this accident, we realised that this method (as well as Rasmussen’s Risk Framework [11], which underpins AcciMap and the *Generic AcciMap Method*) does not have an explicit way to represent Information and Communication Technology (ICT) aspects. ICT is a complex, largely non-physical and critical part of the service delivery infrastructure of a telecommunications operator, encompassing information, data, applications and infrastructure, so it should get special attention and be explicitly represented. Without explicit representation of ICT in the diagrams crucial information is deemphasized and obscured. Therefore, we added an ICT layer to the method to describe the consequences of an attack on digital components and the role they played in the eventual breakdown of the service. We also noticed that we had to spend some time explaining the objective of the method, which is to find out what actually happened, and *not* to assign blame. To avoid this discussion and to set the minds right, we added a new step to the process in which we first establish the physical (core) accident, which encompasses the technical events that can be described without human actions. This new step led to a better and more constructive atmosphere in the group of experts conducting the analysis.

As a further extension, we realised that we could split the accident into two parts: the *onset of the accident* (“how did it happen?”) and the *resolution* (“how did we get back to business as usual?”). Partitioning the accident into logical phases helps divide the workload of the analysis: we could divide the experts who apply the method to analyse the accident into two groups that could then work in parallel, resulting in a more efficient process.

The accident itself led to a crisis within the organisation, since it disrupted the regular business processes and services offered to the customers. Resolving the accident required a coordinated effort to execute unplanned actions across multiple parts of the organization, which was done by applying crisis management. Both the onset and the resolution are important parts of the accident, due to the following reasons:

*Onset*: Understanding the onset can reveal underlying weaknesses in the organization. By strengthening those weaknesses companies can prevent future accidents.
*Resolution*: Being able to quickly and adequately resolve the accident and the ensuing crisis can shorten the time needed to get back to normal operation and therefore compensate or mitigate the impact of the accidents.

The particular accident investigated in the first iteration was in part caused by a positive feedback loop that could not be represented using AcciMap (nor using the *Generic AcciMap Method*). Therefore, we introduced new notation to represent this kind of loop in an AcciMap diagram. All these improvements led to the first version of the *Telecommunications-Related AcciMap Method* (Tram).

#### Second Iteration

**Fig. 2:**
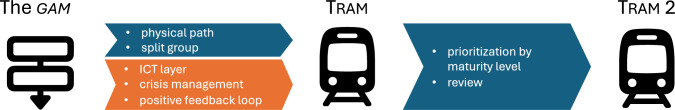
Evolution of Tram, starting from the *Generic AcciMap Method*. The improvements to the process are in the blue arrows, the improvements to the method are in the orange arrow

We then applied Tram’s first version to a new case study in which we analysed a roaming outage caused by a malfunctioning power switch. This resulted in a partial validation of the improvements we had introduced: splitting the groups, describing the physical path of the accident first, adding the ICT layer and applying the notation for the positive feedback. We were unable to validate the application of crisis management since the company resolved the accident smoothly and quickly. As the number of recommendations from the previous applications of Tram was high (136 and 63, respectively), we wanted to help the company in prioritising the different recommendations. Therefore, we further improved the method by adding a strategy to prioritise the recommendations that were formulated using the method, and we improved efficiency by having the group review the diagram between workshops. We called this new version Tram 2. Figure 2 summarises the evolution steps that resulted in the current version of Tram (Tram 2).

In Section Validation of Tram 2, we discuss the case study with which we further validated Tram 2.

### Method Overview

**Fig. 3:**
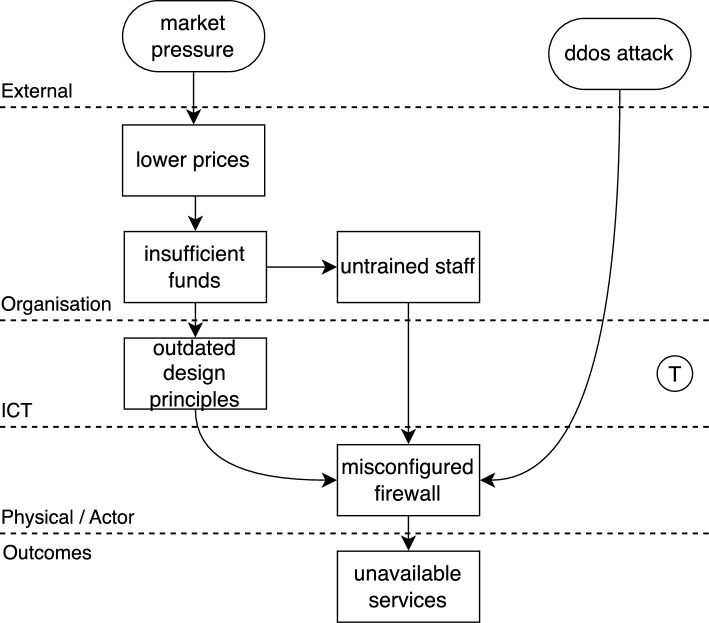
Tram diagram for a fictitious accident (from [4])

Figure 3 is an example of a Tram diagram (our version of the AcciMap diagram) showing how actions or failures culminated in an accident. The symbol  in the figure and in the text that follows indicates the facilities that we have added to the *Generic AcciMap Method* when developing Tram.

To apply Tram, workshops should be organised with subject matter experts from the organisation in which the accident has occurred. The accident is then analysed according to the following steps:

1. *Prepare the Analysis Workshop.*: Available documentation to prepare the analysis workshops is studied, and a list of participants is drafted.
2. *Identify the Physical Path.*
Technical failures that caused the accident are identified and a diagram representing these technical failures is drawn. This step aims to form a common picture among the participants in factual terms, preventing the ‘blame game’.
3. *Split the Accident and the Group.*
The accident can sometimes be split into two distinct phases, such as (a) the onset of the actual accident (*e.g.*  a short-lasting power outage and a subsequent longer service outage), and (b) the resolution of the crisis situation caused by the accident. If this is the case, the group of experts should be split so that each phase of the accident is analysed by the staff involved in that phase.
4.*Identify the Outcomes.*: The consequences of the accident are identified. These may be detrimental, but also beneficial, such as using the crisis to implement long-delayed improvements.
5. *Identify the Causal Factors.*: All causal factors (the nodes in the causal chain of events, represented by the boxes in the diagram) are identified. In this step, the group should not limit itself to actual events, but also take into account measures that were taken but failed to stop the development of the accident or mitigate its consequences.
6. *Identify the Appropriate Layer.*
A Tram diagram is drawn, with layers for External, Organisational, ICT and Physical/Actor causal factors. Each causal factor is plotted in a layer.
7. *Bring the Groups and the Diagrams Together.*
If the group has been split in step 3, the groups are now recombined, to review each other’s diagrams and unify them.
8. *Fill Gaps and Check Logic.*: The group steps through the complete causal chain, identifying missing causal factors and faulty links to finalise the diagram.
9. *Formulate Recommendations.*: For each causal factor, the group discusses how to *prevent* those factors from happening, how to *control* them if they are happening, and how to *compensate* or *mitigate* the consequences once the factor has happened. These recommendations must be formulated in actionable form as they are the output of the analysis.

When developing Tram, we considered improvements at two levels, namely (a) Method level and (b) Process level. We added steps and layers as indicated above (with ), but we also defined additional notation to indicate positive feedback loops, and heuristics to critically consider Crisis Management after the accident occurred. This resulted in a 25% more efficient approach by splitting the group and avoiding discussions about blame, as well as a more effective approach by clearly indicating ICT aspects and by adding Crisis Management to the analysis [3]. We have a total of 7 improvements in the evolution of Tram. We already discussed four of them in our previous paper [3]:

i. starting with the description of the physical path, thus avoiding the blame game;
ii. splitting the group according to the parts of the accident;
iii. adding the ICT layer; and
iv. adding crisis management analysis to the method.

### Improvements

This section discusses in more detail some key improvements we made to the *Generic AcciMap Method* in order to eventually come up with Tram 2.

#### Model Improvement: Positive Feedback Loops

**Fig. 4:**
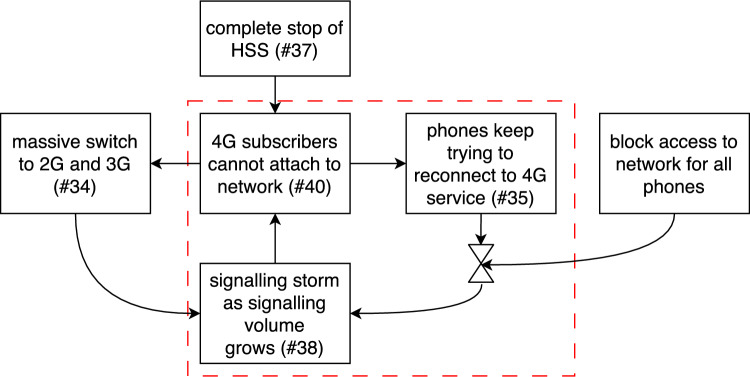
Feedback loop that was instrumental to properly describe the accident discussed in Section Validation of Tram 2 using the notation introduced in [3]. Numbers refer to the numbers in Fig. 5 (from [4])

Since cause-and-effect situations are represented in Tram diagrams, in essence these diagrams are directional non-circular graphs. In [3], we noticed that a positive feedback loop was instrumental in causing an accident. However, it is quite difficult to adequately represent these loops in a strict cause-and-effect diagram, because this either requires a repetitive string of actions, or the positive feedback loop has to be modelled as a single event, with the drawback that the parts that make up the loop and the way to break it are not properly modelled. To cope with these issues, we introduced new notation to represent these loops. The non-circularity requirement has been relaxed, so that we can properly represent each feedback loop with enough detail to show where the loop could be interrupted. We used a valve symbol ($\bowtie$) to indicate where the loop was interrupted and by which action. Figure 4 shows an example from our case study that illustrates this notation. The feedback loop is highlighted with a dashed red box; the action ’*block access to network for all phones*’, which is represented with the square connected to the arrow pointing to the middle of the $\bowtie$ symbol, breaks the feedback loop, and the $\bowtie$ symbol itself indicates the link that gets cut when the loop is broken.

#### Method Improvement: Prioritising Recommendations

In earlier applications of the method [2, 3], a large number of recommendations were generated (136 recommendations in Case Study 1 and 63 recommendations in Case Study 2). Consequently, some guidelines became necessary to help choose which recommendations to implement and in what order, since these choices are far from trivial. For this reason, we investigated in the current case study if we could prioritise the recommendations by using a maturity model.

A number of maturity models are described in the literature [16–20] and a few of them describe how to build maturity models, such as [21]. Six different maturity model development processes were identified in [18] , while [20] describes 21 different maturity models for 6 different domains, such as Business Intelligence, Human Resources and Software Development/Technology. Maturity models typically have two dimensions [22]: (a) process areas, capabilities or characteristics of the domain, and (b) the maturity achieved by the organisation in one of the process areas or capabilities in the first dimension. We prescribe the use of discrete levels for the maturity dimension rather than a continuous scale to enable participants in the case study to more easily categorize and prioritize the various recommendations. The majority of the maturity models in [20] (13 out of 21) and in [22] (8 out of 11) use 5 levels of maturity, so we also used 5 levels.

By giving descriptions to the different levels, the participants have heuristics to assign recommendations to a level. We based the description of our levels on [22]. In this study, the authors identify eleven models, of which four use the same scales, while the other seven use distinct scales. Three models in [20] use the same scales as the four in [22]. These maturity models have been defined for the Software Development/Technology domain. All other maturity models in [20] use distinct names for the levels. The common scales are shown in Table 1, but we adapted the descriptions to make them more generally applicable. We also included examples from a telecom-specific context in which a telecom operator handles customer complaints.

**Table 1:** Common scale in Maturity Models, adapted from [22] (from [4]).

Stage	Description
1—Initial	No processes exist; work is done according to the individual’s own preferences and approaches differ between applications.**Example**: there is no defined complaint procedure. Complaints arrive per mail or per phone at the helpdesk and individual employees pick up the complaint to handle it
2—Repeatable	Work is done according to a repeatable approach which exists in the employee’s mind. **Example**: the company has a tool to register complaints, to helps the employees retrieve important information about the complaints. The individual employee handles the complaints according to her own way of work
3—Defined	Work is done according to a documented procedure. **Example**: the employees work according to a manual; the employees process complaints according to an agreed way of work and all employees follow the steps prescribed in the manual
4—Managed	Work is done according to a documented procedure and the procedure is evaluated and adapted on a frequent basis to improve efficiency and adaptability to changes in the environment of the work. **Example**: all complaints are processed according to the documented procedure. Every year, the helpdesk team sit together to evaluate the procedure and to change steps if that makes the work more efficient or easier, while maintaining the quality of the complaint procedure
5—Optimising	Data is gathered and used to improve the efficiency and effectiveness of the procedures. **Example**: The lead time for processing complaints , the number of complaints and the severity of the complaints are registered. During the day, these numbers are made visible and management can add resources if processing falls behind. The numbers are also used in the yearly evaluation to find spots where the procedure needs to be improved if, e.g., a specific type of complaint becomes common or is time consuming to process

To further simplify the prioritization process, we decided to group the recommendations into clusters and focus on prioritizing within each cluster. For this purpose, staff from the telecommunications operator and researchers are tasked to cluster the recommendations based on their respective business functions. We named the resulting clusters according to the business function and we called these functions *capabilities* an example of this is 2. The subject matter experts are then expected to prioritize the various recommendations, considering that certain recommendations may need to be implemented before others. All recommendations should then be mapped on the maturity scale of Table 1, and that defines the priority for implementing each recommendation.

#### Process Improvement: Offline Review of Diagrams

We also realised that designing the diagrams can be quite labour intensive, since in our previous case studies [2, 3] the participants took 2 or 3 workshops to complete all 9 steps. We observed that efficiency is improved by documenting the diagrams using a drawing tool and sending digital copies out for review between the consecutive workshops. This enhancement improves quality by introducing an additional offline review round, while also boosting efficiency by allowing remarks to be processed offline. This, in turn, avoids consuming valuable time from the subject matter experts.

## Validation of Tram 2

Given the sensitive nature of the accident, we have used the pseudonym *Gamma* in place of the company’s actual name. Furthermore, specific details about the incident have been omitted to maintain the company’s anonymity.

### Accident Cause: Congestion

Gamma is provider of GSM mobile services. In 2018, Gamma virtualized a critical network component, namely a Signal Transfer Point (STP), which is a routing device used to set up phone calls. This device had been functioning smoothly until Gamma needed to implement a configuration change. To prepare for this update, Gamma created a snapshot (backup) of the virtualized STP, allowing for a rollback if any issues arose. However, while the snapshot was being created, the virtual STP temporarily became unresponsive. During this downtime, another network element—the Home Subscriber Server (HSS), which manages subscriber data and service information—sent traffic to the STP that the STP could not acknowledge. This led to a buffer overrun in the HSS, ultimately causing it to cease functioning. As a result, subscribers lost access to the 4G network, with their devices automatically switching to 2G and 3G networks while simultaneously trying to reconnect to 4G. This triggered a signaling storm that overwhelmed the HSS and led to a complete service outage.

**Fig. 5:**
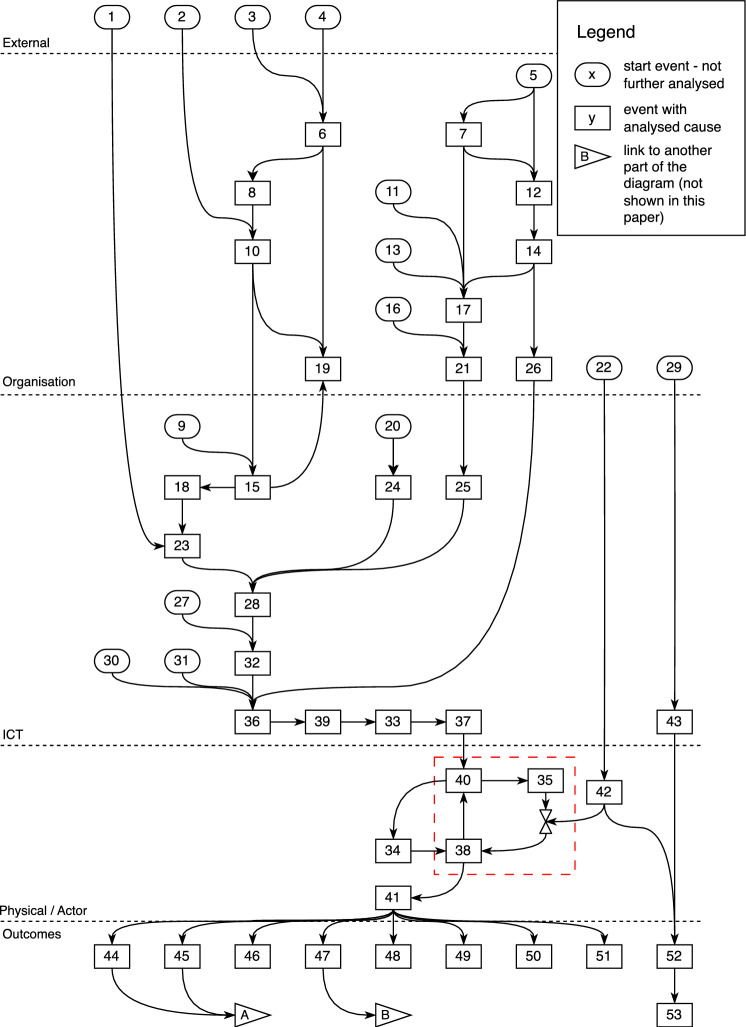
Tram diagram of the technical part of the accident (from [4])

The issue was resolved by Gamma by blocking network access for all phones and restarting the HSS after that. The service was restored by releasing the phones in small batches in a controlled way. Figure 5 shows the Tram diagram of the accident. To further protect the identity of the company, we have used numerical labels in the boxes in the figure instead of descriptive text. In Fig. 5, the positive feedback loop (4# 40 – # 35 – # 38 –# 40) caused the congestion and the network breakdown (# 41). The triangles with *A* and *B* indicate connections to the diagrams that represent the consequences, which are omitted in this paper for the sake of space and because they do not give any additional insights.

### Consequence: Crisis

For budgetary reasons, Gamma had not established a centralised crisis communication organization. Furthermore, adequate tooling was missing, leaving Gamma unprepared for this crisis. An exacerbating circumstance was that the Gamma staff were using Gamma subscriptions. This meant that during the first part of the crisis, no effective internal communication was possible, as Gamma’s GSM service was down. The staff made an app group on WhatsApp, which was cumbersome and hindered the resolution of the crisis.

As Gamma was the primary telecommunications provider for both government agencies and hospitals in the country, the crisis led to a breakdown in governmental communications and rendered hospitals unreachable. The Ministry of Economic Affairs, informed of the crisis even before Gamma’s internal liaison, immediately held the company accountable, further damaging Gamma’s reputation.

### Results of the Accident Analysis

We organised four workshops to analyse the accident with Tram, two with technical staff and two with communications staff. In these workshops, 91 causal factors were uncovered, leading to 65 outcomes and 265 recommendations that the company could implement. While analysing the accident, we also validated our seven proposed improvements to AcciMap, where four of these improvements aligned with our previous findings in [2, 3]. The remaining three improvements are discussed below.

#### Model Improvement: Positive Feedback Loops

Figure 5 shows that a feedback loop was responsible for the breakdown of the network, and the company could only restore service after breaking this loop. With the notation we introduced in the case study reported in [3], we successfully modelled both the feedback loop and how it has been broken. By modelling at this level of detail we could issue recommendations for both preventing and breaking the feedback loop. In this process, our notation was both justified and validated.

#### Method Improvement: Prioritising Recommendations

A follow-up workshop with the company’s business continuity managers was organised after the recommendations were formulated. In this follow-up workshop, the recommendations were clustered as shown in Table 2.

**Table 2:** Clusters of recommendations (from [4])

Cluster description	# recommendations
Manage Communications	107
Manage Crisis	81
Manage Growth and Maturity	16
Manage Problem	10
Manage Reporting	1
Manage Resilience	44
Manage Resolution of Situation	6

The business continuity managers have shared the clustered recommendations with the participants in the workshops and to order the recommendations according to their *dependencies* (which recommendations could be implemented right away, and which were dependent on other recommendations that needed to be implemented first) and *urgency* (which recommendations needed to be implemented as soon as possible and which could wait). With this information, we could have plotted the recommendations on the scale shown in Table 1. However, unfortunately, the business continuity managers could not motivate the participants to complete this task, so these specific results could not be achieved. For the same reason, costs and benefits could not be formulated either, illustrating one of the difficulties frequently encountered when applying Technical Action Research.

We propose that this unresponsiveness from the participants could have been avoided by ensuring that the analysis had a sponsor at a senior management level. Our intended results have aimed to improve the company’s resilience, thereby helping to assure business continuity. Since the business continuity is a responsibility of the board, senior management sponsorship would have been justified. Furthermore, we assume that senior management can compel employees to cooperate, whereas the business continuity managers can only (politely) ask. To secure such support, future implementations should start with gathering senior management support. This should not be hard, as the method will mostly be applied to large accidents that have already had senior management support during the resolution phase of the accident. If senior management is not interested in sponsoring the analysis (even though they are compelled to perform such an analysis by law), the effectiveness of the analysis and more specifically the implementation of the recommendations is not guaranteed, and the analysis may then just be a waste of valuable resources.

#### Process Improvement: Splitting the Group According to Expertise

During the Covid-19 pandemic, gatherings were prohibited. Therefore, we performed our workshops online through Microsoft Teams and used Excel to record our results. Efficiency was affected by that since the way these workshops were conducted differed vastly from the previous ones where participants gathered in a room around a brown paper wall. Consequently, we could not assess the efficiency of splitting the groups according to expertise.

In addition, in the workshops we noticed that it was harder to ensure that ‘the other side’ (operations versus communications) was not blamed. The sharing of ideas between people from different areas of expertise was not observed in the workshops, unlike what happened in our previous case studies where the interaction led to a common goal of learning from the accident on a broader scale than just within individual departments.

## Discussion

In this section, we summarise the main lessons of our case study.

### Feedback Loop

Introducing a notation to represent feedback loops brought two main benefits:

1. We can now represent one of the material causes of an accident, bringing more insight into the development of the accident and possible ways to prevent a feedback loop from occurring at all.
2. We can now represent how the positive feedback loop can be broken, giving valuable recommendations for future accidents in which feedback loops play a role. Identifying its solution enables the organisation to draw up step-by-step instructions or action plans for managing this type of situation.

### Prioritisation

Since we never received feedback from participants, unfortunately, we could not prioritise the recommendations. The involvement of senior management to instruct staff to cooperate with the analysis could have helped (see Section Method Improvement: Prioritising Recommendations). However, by identifying clusters of recommendations, we were able to identify candidates for capabilities that could be part of a maturity model. We can argue that the prioritisation was partially validated, but this preliminary result still needs to be fully validated in follow-up case studies.

### Splitting the Group According to Expertise

A quantitative comparison of the meetings’ efficiency was not possible due to the impact of the Covid-19 pandemic. However, we could observe that splitting the group along the lines of expertise had a negative impact since it removed a valuable aspect of the previous case studies, namely the sense of a common goal between different departments. Furthermore, we could not achieve an atmosphere in which the principle of ‘no blame’ could prevail. We have observed unequal participation; it was hard to ensure equal participation during the online meeting. The body language was only partially visible, which means that signals may have been missed by people who tended to disagree but did not express their disagreement vocally. Additionally, the effort may have been perceived as less collaborative than when participants were physically in the same room, resulting in a more individualised approach rather than a cohesive team effort, which could have affected trust levels and candour among participants.

This implies that, because we conducted this case study under different circumstances than in our previous study [3], we cannot draw many general conclusions from this observation. However, it is worth noting that virtual collaboration tools and techniques can foster a collaborative culture in meetings. Integration of online tools and techniques can be a future improvement in Tram 2.

WE also concluded that online participation can always hinder collaboration in such a sensitive environment, as [23] highlights. Online settings usually miss the broader focus of face-to-face meetings and the trust component. In the case of telecom companies, if the company already has a culture of trust, then an online workshop can achieve the desired outcomes. We can consider this an open research direction, exploring how online workshops affect trust and how existing trust levels impact workshop outcomes.

### Threats to Validity

The results of our study were obtained from a single case study (two case studies for the notation of positive feedback loops), which poses a threat to their validity. The capabilities for telecommunications business continuity have been drawn from recommendations following the analysis of a specific accident. We foresee that other accidents may yield different capabilities.

The workshops were conducted in unusual circumstances —online due to Covid lockdowns —adding to the threat to validity. Therefore, we were unable to distinguish between the impact of online working and the effect of splitting the group by expertise.

## Final Remarks

This paper highlights the evolution of Tram from the AcciMap method through an iterative development process and also discusses the last iteration in detail, in which the modelling of positive feedback loops, which can play a crucial role in telecommunications accidents, has been validated and in which the prioritisation of recommendations has been added to the method. Moreover, this paper contributes to the state-of-the-art literature discussion by identifying how the rise of systemic methods as noticed in our previous literature review [8] has continued. This literature also shows that AcciMap is the most widely applied systemic accident analysis method, thereby justifying our choice to base Tram on AcciMap.

In our research, we observed that by making positive feedback loops explicit, we could model the risk of triggering a cascade of consequences that could cause even more damage to the organisation. Special attention should be paid to splitting the group by expertise or incident stage. Furthermore, we recommended obtaining support from senior management when applying the method to ensure the organisation’s cooperation.

Further research into capability maturity models to prioritise recommendations by clustering them to help the crisis management team may yield new insights. AS future work, we can do a focused case study with higher management support and have a team of experts go through the recommendations with the purpose of plotting them into the maturity model. Another method to validate the prioritisation mechanism can be via a simulated environment with stakeholder involvement.

## Data Availability

The data for this research is available from the corresponding author upon request. The data will be anonymised where relevant.

## Declarations

### Research Involving Human and/or Animals

All participants voluntarily participated in the workshops in this study. No personal data was used.
